# First Organoid Intelligence (OI) workshop to form an OI community

**DOI:** 10.3389/frai.2023.1116870

**Published:** 2023-02-28

**Authors:** Itzy E. Morales Pantoja, Lena Smirnova, Alysson R. Muotri, Karl J. Wahlin, Jeffrey Kahn, J. Lomax Boyd, David H. Gracias, Timothy D. Harris, Tzahi Cohen-Karni, Brian S. Caffo, Alexander S. Szalay, Fang Han, Donald J. Zack, Ralph Etienne-Cummings, Akwasi Akwaboah, July Carolina Romero, Dowlette-Mary Alam El Din, Jesse D. Plotkin, Barton L. Paulhamus, Erik C. Johnson, Frederic Gilbert, J. Lowry Curley, Ben Cappiello, Jens C. Schwamborn, Eric J. Hill, Paul Roach, Daniel Tornero, Caroline Krall, Rheinallt Parri, Fenna Sillé, Andre Levchenko, Rabih E. Jabbour, Brett J. Kagan, Cynthia A. Berlinicke, Qi Huang, Alexandra Maertens, Kathrin Herrmann, Katya Tsaioun, Raha Dastgheyb, Christa Whelan Habela, Joshua T. Vogelstein, Thomas Hartung

**Affiliations:** ^1^Center for Alternatives to Animal Testing (CAAT), Department of Environmental Health and Engineering, Bloomberg School of Public Health and Whiting School of Engineering, Johns Hopkins University, Baltimore, MD, United States; ^2^Department of Pediatrics and Cellular and Molecular Medicine, School of Medicine, University of California, San Diego, San Diego, CA, United States; ^3^Center for Academic Research and Training in Anthropogeny (CARTA), Archealization Center (ArchC), Kavli Institute for Brain and Mind, University of California, San Diego, San Diego, CA, United States; ^4^Viterbi Family Department of Ophthalmology & the Shiley Eye Institute, UC San Diego, La Jolla, CA, United States; ^5^Berman Institute of Bioethics, Johns Hopkins University, Baltimore, MD, United States; ^6^Department of Chemical and Biomolecular Engineering, Johns Hopkins University, Baltimore, MD, United States; ^7^Department of Chemistry, Johns Hopkins University, Baltimore, MD, United States; ^8^Department of Materials Science and Engineering, Johns Hopkins University, Baltimore, MD, United States; ^9^Laboratory for Computational Sensing and Robotics (LCSR), Johns Hopkins University, Baltimore, MD, United States; ^10^Center for Microphysiological Systems (MPS), Johns Hopkins University School of Medicine, Baltimore, MD, United States; ^11^Oncology and Sidney Kimmel Comprehensive Cancer Center, Johns Hopkins University School of Medicine, Baltimore, MD, United States; ^12^Department of Biomedical Engineering, Johns Hopkins University, Baltimore, MD, United States; ^13^Janelia Research Campus, Howard Hughes Medical Institute, Ashburn, VA, United States; ^14^Departments of Biomedical Engineering, Carnegie Mellon University, Pittsburgh, PA, United States; ^15^Department of Materials Science and Engineering, Carnegie Mellon University, Pittsburgh, PA, United States; ^16^Department of Biostatistics, Johns Hopkins Bloomberg School of Public Health, Johns Hopkins University, Baltimore, MD, United States; ^17^Department of Computer Science, Whiting School of Engineering, Johns Hopkins University, Baltimore, MD, United States; ^18^Department of Physics and Astronomy, Krieger School of Arts and Sciences, Johns Hopkins University, Baltimore, MD, United States; ^19^Mark Foundation Center for Advanced Genomics and Imaging, Johns Hopkins University, Baltimore, MD, United States; ^20^Department of Statistics and Economics, University of Washington, Seattle, WA, United States; ^21^Department of Ophthalmology, Johns Hopkins University School of Medicine, Baltimore, MD, United States; ^22^Department of Molecular Biology and Genetics, Johns Hopkins University School of Medicine, Baltimore, MD, United States; ^23^Department of Genetic Medicine, Johns Hopkins University School of Medicine, Baltimore, MD, United States; ^24^Department of Neuroscience, Johns Hopkins University School of Medicine, Baltimore, MD, United States; ^25^Department of Electrical and Computer Engineering, Johns Hopkins University, Baltimore, MD, United States; ^26^Department of Research and Exploratory Development, Johns Hopkins University Applied Physics Laboratory, Laurel, MD, United States; ^27^Philosophy Program, School of Humanities, University of Tasmania, Hobart, TAS, Australia; ^28^AxoSim Inc., New Orleans, LA, United States; ^29^Luxembourg Centre for Systems Biomedicine (LCSB), University of Luxembourg, Esch-sur-Alzette, Luxembourg; ^30^School of Biosciences, College of Health and Life Sciences, Aston University, Birmingham, United Kingdom; ^31^Department of Chemistry, School of Science, Loughborough University, Loughborough, Leicestershire, United Kingdom; ^32^Department of Biomedical Sciences, Institute of Neuroscience, University of Barcelona, Barcelona, Spain; ^33^Clinic Hospital August Pi i Sunyer Biomedical Research Institute (IDIBAPS), Barcelona, Spain; ^34^Department of Molecular and Comparative Pathobiology, Johns Hopkins University, Baltimore, MD, United States; ^35^Aston Pharmacy School, College of Health and Life Sciences, Aston University, Birmingham, United Kingdom; ^36^Department of Biomedical Engineering, Yale Systems Biology Institute, Yale University, New Haven, CT, United States; ^37^Department of Bioscience and Biotechnology, University of Maryland Global Campus, Rockville, MD, United States; ^38^Cortical Labs, Melbourne, VIC, Australia; ^39^Department of Neurology, Johns Hopkins School of Medicine, Baltimore, MD, United States; ^40^Center for Alternatives to Animal Testing (CAAT)-Europe, University of Konstanz, Konstanz, Germany

**Keywords:** microphysiological systems, brain, electrophysiology, cognition, artificial intelligence, biological computing, Organoid Intelligence

## Abstract

The brain is arguably the most powerful computation system known. It is extremely efficient in processing large amounts of information and can discern signals from noise, adapt, and filter faulty information all while running on only 20 watts of power. The human brain's processing efficiency, progressive learning, and plasticity are unmatched by any computer system. Recent advances in stem cell technology have elevated the field of cell culture to higher levels of complexity, such as the development of three-dimensional (3D) brain organoids that recapitulate human brain functionality better than traditional monolayer cell systems. Organoid Intelligence (OI) aims to harness the innate biological capabilities of brain organoids for biocomputing and synthetic intelligence by interfacing them with computer technology. With the latest strides in stem cell technology, bioengineering, and machine learning, we can explore the ability of brain organoids to compute, and store given information (input), execute a task (output), and study how this affects the structural and functional connections in the organoids themselves. Furthermore, understanding how learning generates and changes patterns of connectivity in organoids can shed light on the early stages of cognition in the human brain. Investigating and understanding these concepts is an enormous, multidisciplinary endeavor that necessitates the engagement of both the scientific community and the public. Thus, on Feb 22–24 of 2022, the Johns Hopkins University held the first Organoid Intelligence Workshop to form an OI Community and to lay out the groundwork for the establishment of OI as a new scientific discipline. The potential of OI to revolutionize computing, neurological research, and drug development was discussed, along with a vision and roadmap for its development over the coming decade.

## Overall scope of the workshop

Advances in stem cell culture and bioengineering research have increased the complexity of cell cultures toward *in-vivo*-like physiology and architecture, a platform known as microphysiological systems (MPS) (Marx et al., 2016, 2020). In the case of brain MPS, these improvements create new possibilities for modeling cognition as synthetic biological intelligence and introduce a promising new field of research that we coin Organoid Intelligence (OI) (Smirnova et al., 2023). This is an interface between living tissue and computer technology whereby brain cell cultures grown into 3D structures, also known as organoids, are integrated into organ-on-chip systems, and the resulting output data is interpreted. Feeding input information and feedback to the output information, simple sensing, and processing of information must all be realized. A key question will be, to what extent will the brain organoids be able to adapt and memorize, i.e., learn. By leveraging the organoid's brain-like functionality, we can harness its capacity to process complex inputs, study of the physiology of learning, and generate responses to control peripheral output devices.

The human brain processes information extremely efficiently and is unmatched by modern computers, both in terms of data processing and energy efficiency. The potential for brain organoids to perform as biological computers is being explored by implementing a combination of electrophysiology, high-content imaging, AI (Artificial Intelligence) for pattern recognition, and brain/sensory organoid and brain/machine interfaces to identify biological computing's challenges and opportunities.

The first Organoid Intelligence workshop, forming an OI Community, organized by Johns Hopkins University Feb 22–24 of 2022, served as a starting point for the establishment of OI as a recognized emerging scientific discipline through the Baltimore Declaration toward OI (Hartung et al., 2023; Box 1). Members of the global scientific community came together to present, listen, compare notes, and herald a new, multidisciplinary field of science and engineering. OI's potential to revolutionize computing was discussed, along with a vision for its development over the coming decade, the potential contributions to neurological research and drug development, and the important ethical considerations that it entails.

Box 1 **The Baltimore Declaration toward the exploration of organoid intelligence (Hartung et al., 2023).**

The Baltimore Declaration toward the exploration of organoid intelligence We the participants of the First Organoid Intelligence Workshop–“Forming an OI Community” (22–24 February 2022), call on the international scientific community to explore the potential of human brain-based organoid cell cultures to advance our understanding of the brain and unleash new forms of biocomputing while recognizing and addressing the associated ethical implications. The term “organoid intelligence” (OI) has been coined to describe this research and development approach (Smirnova et al., 2023) in a manner consistent with the term “artificial intelligence” (AI)–used to describe the enablement of computers to perform tasks normally requiring human intelligence. OI has the potential for diverse and far-reaching applications that could benefit humankind and our planet, and which urge the strategic development of OI as a collaborative scientific discipline. OI holds promise to elucidate the physiology of human cognitive functions such as memory and learning. It presents game-changing opportunities in biological and hybrid computing that could overcome significant limitations in silicon-based computing. It offers the prospect of unparalleled advances in interfaces between brains and machines. Finally, OI could allow breakthroughs in modeling and treating dementias and other neurogenerative disorders that cause an immense and growing disease burden globally. Realizing the world-changing potential of OI will require scientific breakthroughs (Smirnova et al., 2023). We need advances in human stem cell technology and bioengineering to recreate brain architectures and to model their potential for pseudo-cognitive capabilities. We need interface breakthroughs to allow us to deliver input signals to organoids, measure output signals, and employ feedback mechanisms to model learning processes. We also need novel machine learning, big data, and AI technologies to allow us to and understand brain organoids. In addition to confronting these scientific and technical challenges, we also need to anticipate (as far as possible) and address the significant and largely unexplored ethical challenges associated with this research. We must be alert to any possibility that organoids could develop forms or aspects of consciousness and mitigate and safeguard against this. The cell donor's personal rights and interests are among other important considerations. These issues warrant stringent, ongoing discussions throughout the development of OI with the aim of producing an accepted ethical framework. Such discussions should include all relevant stakeholders and take due account of public values. We are only just beginning this multidisciplinary and multistakeholder endeavor. The potential benefits are world-changing, but the challenges are daunting. We call on the scientific community to join us on this journey. Only by collaborating will we be able to realize the full potential of OI to advance science, technology, and medicine.

### Opening remarks

Fred Fenter, Chief Executive Editor of Frontiers, the peer-reviewed, open-access science publisher based in Lausanne, Switzerland, opened the workshop by explaining that the mission of Frontiers' new flagship journal, Frontiers of Science, is to “bring an understanding of the most important scientific research to all members of society.” The strategy, he explained, is to build an environment around each article and create communication channels to translate the implications of the work being published to all members of society, from kids and their families, to the scientific community at large. “In this journal, there will be scientific perspectives written by experts in the fields, editorials written by opinion leaders, summaries, and infographics for the non-science community, and a children's version of the article.” Fenter said this strategy will be used to support the dissemination of the organoid intelligence project with a perspective article (Smirnova et al., 2023), which was shared as an early draft with the participants before the workshop, as an inaugural article.

Thomas Hartung, Doerenkamp-Zbinden-Chair and Professor of Evidence-Based Toxicology at Johns Hopkins, Director of the Center for Alternatives to Animal Testing (CAAT), and field chief editor for Frontiers in AI, discussed the concept of Organoid Intelligence (OI) and how, over the last 2 years, the Hopkins's team has been shaping the idea of OI and developing it using four separate tracks: ethics, organoids, electrophysiology, and computational data processing.

Hartung outlined OI's potential use as a biological computer and how to make brain cultures more like a computer. He described how, in 2001, Steve Potter and colleagues at Georgia Tech used cultured 2D rat cells to control a robot and have it explore its environment (Demarse et al., 2001). Now, in 2021, as a proof-of-concept of intelligence and trainability using 2D cultures, colleagues from Cortical Labs (also workshop participants) are able to train neurons to play pong (Kagan et al., 2022). Hartung highlighted the improvements that bioengineering and stem cell technology have had on MPS such as organ-on-chip, scaffold-based 3D tissue, organoids, and organ/human-on-chip, as well as their collective potential for improving drug development. Another milestone for MPS came with the development of a guidance document on Good Cell and Tissue Culture Practice 2.0 (GCCP 2.0) (Pamies et al., 2020). All this was presented at the MPS World Summit, which took place between May 30 and June 3, 2022,^1^ and served as a platform to present for the first time the OI concept. Hartung discussed the concept of intelligence-in-a-dish and how learning is expected to change the organoid. “Can we grow the next supercomputer in a lab?,” Hartung asked provocatively. Lastly, Hartung elaborated on how “learning-in-a-dish” might be applied to study conditions such as Asperger's, impaired neurodevelopment, and dementia, and how to do it ethically, stressing the importance of “embedded ethics” in the OI project.

## Ethics track introduction

Harnessing the innate biological capabilities of brain organoids for biocomputing and synthetic intelligence raises complex questions about organoid consciousness and sentience. Emerging technologies related to the use of organoids, particularly neural or cerebral organoids, require strict ethical frameworks, and practices and guidelines should reflect the values and attitudes of an informed public.

Jeffrey Kahn briefly discussed his work as co-chair of the neuroethics working group for the NIH BRAIN 2.0 strategic plan in October 2019. Jeffery Kahn is the Andreas C. Dracopoulos Director of the Johns Hopkins Berman Institute of Bioethics, where he also holds the Robert Henry Levi and Ryda Hecht Levi Professorship in Bioethics and Public Policy. He is also a professor in the Department of Health Policy and Management of the Johns Hopkins Bloomberg School of Public Health. Kahn then introduced his colleagues Deborah Mathews and Lomax Boyd to talk about their work in the area of brain organoid ethics.

Debra Mathews, Assistant Director for Science Programs at Johns Hopkins Berman Institute of Bioethics and Associate Professor at the Department of Genetic Medicine at Johns Hopkins University presented the results of a recent pilot project Stakeholder Attitudes Toward Organoid Research Investigation (SATORI), that aimed to systemically obtain data from a set of patient attitudes related to organoid research where clinical translation from this platform is realistically expected (Bollinger et al., 2021). An additional aim sought to develop ethical guidance for the clinical translation of these scientific tools. In this pilot project, 60 interviews were conducted with individuals with cystic fibrosis, gastrointestinal, neurological, or eye disease, as well as with general outpatients. The study included fair gender representation and diverse ethnicities. Educational levels were skewed toward highly educated, with one-third holding graduate degrees and a second third with college degrees. The results showed broad support for the derivation and use of organoids for their potential to advance human health and drug discovery. However, brain organoids elicited a conditional response in which stakeholders more seriously considered the intent of the research and concluded that there should be boundaries in place to limit ethically questionable research and its applications. Most importantly, it recommends a thorough process of consent and re-consent, and continual evaluation throughout the scope of the research, particularly if biospecimens were obtained from minors.

J. Lomax Boyd, Assistant Research Professor at the Johns Hopkins Berman Institute of Bioethics, explores the ethical and moral implications of neuroscience research investigating human neurobiology. He introduced a framework for exploring responsive pathways for engaging the public at the early stage of OI technology. He proposed that responsible public engagement with scientific research should include three elements: definitional clarity of the science performed, awareness of cognitive processes underlying moral judgements, and knowledge of how cultural beliefs intersect with science. Dr. Boyd has served as a Civic Science Fellow in partnership with the Kavli Neuroscience Discovery Institute, and did his postdoctoral training in the Laboratory of Neurobiology of Speech and Language at Rockefeller University.

## Brain organoids track introduction

Alysson Muotri, a professor in the Department of Pediatrics and Cellular and Molecular Medicine at the University of California, San Diego, presented the development of human brain organoid technology to date. He remarked on the importance of understanding how the brain develops and works under normal conditions, how the brain changes during disease, and what the current limitations are to advancing our understanding. One such limitation is that despite our cumulative knowledge of the brain, we still do not totally understand how it forms and works; therefore, it is difficult to model. Human brain organoids provide a novel tool to study and understand the human brain, and Muotri has made a substantial contribution to this field through the optimization of the protocol to generate functional cortical organoids that exhibit neuro-oscillations. He showed that the oscillatory dynamics in his cortical organoids mimic the bursting phenomenon that happens in pre-natal to post-natal human brain EEGs (Trujillo et al., 2019).

Addressing the question of how biology inspires or can even become part of AI, he explained, “*Contrary to the complexity of cell populations and the dynamic connectivity observed in the real brain, current AI technology uses simplified homogeneous static neurons to build a network. It is likely that the incorporation of cortical organoid circuitry can dramatically improve the current models of AI toward broader aspects of cognition, especially if the goal is to mimic human-like ability and rebut AI algorithms*.”

Muotri's lab has explored the natural intelligence and connectivity of brain organoids and AI by “teaching” a robot to walk and navigate its environment by using the electrical activity coming from the neural oscillations of the organoids (Trujillo et al., 2019), video footage can be accessed here.

Karl Wahlin, Assistant Professor in the Department of Ophthalmology at the University of California, San Diego, and Director of the Richard C. Atkinson Lab for Regenerative Ophthalmology, presented “*Retinal Organoids–human models of the sensory nervous system*.” Wahlin introduced the history, development, and physiology of retinal organoids, as well as some of their applications, emphasizing that it is a well-characterized sensory model, and has a refined time-course of development, beginning as an eye field, optic cup, and eventually evolving into a well-organized retina (Vielle et al., 2021).

“*This is a workshop on brain OI, and so obviously the brain is the most important organ you have, except it is not always the most intelligent organ that you have, unless it has input... It is the input that makes the brain work…sight, hearing, taste, touch, smell: these are all things that organoids really need to develop in order to develop a higher level of connectivity*.” Posing the question as to whether retina organoids exhibit light-sensitive properties, Wahlin cited Giorgia Quadrato (Quadrato et al., 2017) who demonstrated that exposing organoids to light results in electrophysiological responses, and when performing gene expression analysis, organoids showed typical genetic profiles of retinal cells including photoreceptors.

Addressing how input can be given to brain organoids, Wahlin, in collaboration with Alysson Muotri and Shaochen Chen, discussed how they are developing a human visual circuit where they integrate cortical, thalamus, and retinal organoids on scaffolds with biomimetic properties. In principle, this circuit may allow for the exploration of how sensory inputs influence brain development in the near future.

**Figure 1:**
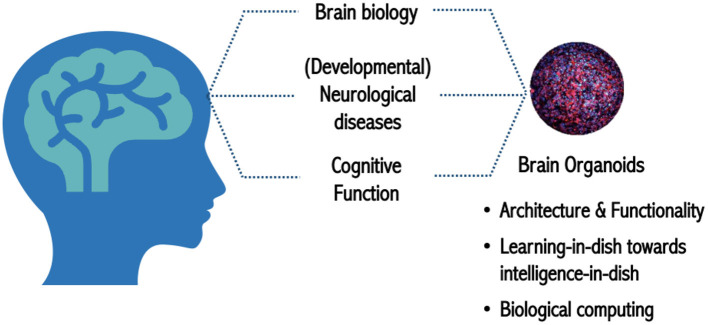
Brain organoids as a model to study human biology in health and disease.

Lena Smirnova, Assistant Professor from CAAT at Johns Hopkins University and director of Education and MPS and Systems Toxicology programs at Hopkins, then addressed the topic of “*What do we need from biology to tackle OI?*.” She restated that brain organoids are part of the MPS and micro(patho)physiological systems and highlighted the importance of applying OI to address neurodegeneration and neurodevelopmental disorders, such as Parkinson's, Alzheimer's, autism, and attention deficit hyperactivity disorder (ADHD) (Figure 1).

Smirnova provided a concise overview of how OI came about, with its origins derived from the BrainSphere model developed at CAAT laboratory (Pamies et al., 2017). This organoid model is reproducible in size and cellular composition of neurons, astrocytes, and oligodendrocytes. Also present is myelination, a key feature of the electrical activity displayed in the model (Chesnut et al., 2021a,b).

In preparation for OI, Smirnova's laboratory has enriched the population of astro- and oligodendro-glia (necessary for maintenance of neuronal networks), and it is in the optimization phase (unpublished observations) for increasing synaptogenesis and incorporating micro-glia–the immune cell of the brain, also responsible for synapse pruning during development.

Smirnova showed how, oligodendrogenesis can be quantified in response to different stimuli using a CRISPR-Cas9-engineered GFP-PLP1 reporter line (Romero et al., 2023). Smirnova's group is currently working on the implementation of a microfluidic system to increase perfusion and biomass (for energy efficiency in biocomputing), as well as biocomplexity.

## Special address by Ed Schlesinger

Ed Schlesinger, the Benjamin T. Rome Dean at Johns Hopkins University's Whiting School of Engineering, welcomed the multidisciplinary and international attendance of the OI workshop and expressed his excitement and interest, stating, “*from the creation of brain organoids themselves to understanding the nature of their function, interfacing with [other] organoids and, at some point, understanding the origin of intelligence—that would be quite remarkable*.”

He conveyed that ultimately it is about improving the lives of people, and through advances in OI “*there is the possibility of a global impact*.” He went on to say that this technology will enable new ways of brain/machine interactions, and that the opportunities are mind-boggling. “*This platform can be used to test new hypotheses, predict better clinical outcomes and better therapeutics. While these are efforts actively pursued across Hopkins and other institutions, OI brings an entirely new set of tools to the table, and Johns Hopkins is uniquely suited to the undertaking*.”

He celebrated that colleagues from other institutions are drawing on their collective knowledge and expertise to achieve these objectives. Initiatives like OI “*not only require both broad and deep expertise, it also demands audacious thinking and willingness to take risks...we are thinking big, and the potential is amazing*.”

## Electrophysiology track introduction

David Gracias, professor at Johns Hopkins Whiting School of Engineering with a primary appointment in chemical and biomolecular engineering and secondary appointments in materials science and engineering, chemistry, and oncology, talked about “*Perspectives in integrated platforms for organoid-machine interfaces*.”

“*We envision a system by which we can talk to the living organoid and a computer in a seamless manner back and forth over prolonged periods of time*.” So, what microinstrumentation do we need for OI? What do we need to form a robust connection between a living organoid, which can be small and in aqueous media, and a conventional computer? These were questions posed by Gracias.

In the field of electrophysiology, cells in 2D cultures can be interfaced with planar MEAs (Microelectrode Arrays), but provide only a limited contact area for recording electrical activity. Performing organoid electrophysiology recording in 3D would bring us closer to how human electroencephalography (EEGs) recordings are performed (Huang et al., 2022). However, many challenges arise with attaching electrophysiological devices to organoids which are small and three-dimensional. Such challenges, which include the challenge of high-resolution patterning fidelity in 3D curved geometries required for large I/O interfaces, reliability and reproducibility and the complexity of interconnect packaging from the organoid to the external world, were enumerated. Also, potential solution paths were discussed, such as the use of 3D heterogeneous integration, self-folding and buckling for the incorporation of multi-scale optical, electrical and microfluidic instrumentation platforms by 2D to 3D engineered shape transformation. The road ahead requires strategies to manipulate brain organoids on a chip, and to enable low-noise robust recording, stability, and parallelism. Additional issues involving invasive vs. non-invasive recordings, interconnecting organoids, and *in-situ* control were discussed.

Some of the approaches Gracias is employing are based on lithographically patterned self-folding, electrical, optical and microfluidics fabrics, essentially creating a shell MEA. This shell is like a mini cap analogous to a macroscale human EEG cap (Huang et al., 2022).

The shell MEA for brain organoids has two clear advantages. The first is the high signal-to-noise-ratio, and the second is that it allows for spatio-temporal recording to track electrical activity around the organoid in 3D, which could be of remarkable interest for learning and understanding electrical patterns. David Gracias and collaborator Brian Caffo are using machine learning to analyze the data (Huang et al., 2022).

Gracias envisions electrically and optically active, self-folding, ultrathin film biosensors or atomistic skins for stimulation and recording, as well as curved and folding microfluidics for the generation of chemical gradients and varying the chemical microenvironment around the organoids for OI. Such a multifactorial platform will necessitate infrastructure for microfluidic, optical, and electrical control. The organoids will be able to be interrogated for extended periods, and eventually could be interconnected with other organoids to build significantly complex biological systems. Another aspect of interest is a portable OI system which will require the creation of such an infrastructure in a compact device akin to a suitcase.

John Rogers, a Louis Simpson and Kimberly Querrey Professor of Materials Science and Engineering, Biomedical Engineering and Neurological Surgery, at Northwestern University, and director of the Querrey Simpson Institute for Bioelectronics, then presented his talk, entitled “*3D mesoscale structures as bioelectronic interfaces to cortical spheroids*.” It covered the technical approaches his line of research employs to establish 3D interfaces with small 3D cortical spheroids, muscle tissue, and cardiac spheroids (Park et al., 2021a). These approaches can be used to overcome some of the challenges Gracias enumerated.

One of Rogers' main interests is engineering non-pharmacological approaches to treat disease and disorders. “*Neuromodulation and bioelectronic medicines are more novel and potentially more powerful than traditional medicine*.” In this context, organoids could serve as a powerful platform to understand biological and engineering interfaces, such as electrical, optical, chemical, and thermal, to leverage new forms of bioelectronic medicine (Park et al., 2021a,b).

Technologies presented by Rogers include flexible, hybrid optoelectronics for neural interfaces that allow for embedding light directly onto an area of interest wirelessly. He is also creating 3D mesoscale networks driven by mechanical buckling instabilities, allowing 3D topographical complex structures to grow out of a plane. These structures can be made from varied materials and are fully compatible with integrated circuit technology. In terms of OI electrophysiology, Rogers presented a multifunctional, soft 3D MEA system that provides 3D spatio-temporal mapping of neural activity and can be modified to house multiple organoids to induce organoid inter-communication (Park et al., 2021b). He concluded that the “*future of biological interface devices is soft, multi-functional and 3D*.”

Tim Harris is a group leader and senior fellow at the Howard Hughes Medical Institute (HHMI) Janelia Research Campus, and a Research Professor at Johns Hopkins University Department of Biomedical Engineering. In his talk, “*Considerations for high channel count Si probes as an electrophysiology probe for brain organoids*,” Harris gave a brief overview of recording probes that can track the electrical activity of the same neurons over months. These recording probes called Neuropixels 1.0 and 2.0, may assist in addressing some of the challenges for organoid interfaces. The Neuropixels 1.0 probes work on the basis of low impedance at the recording sites and can stimulate several thousand pulses without degradation (Jun et al., 2017). They have been used in nine species including mice, lizards, marmosets, and humans. The Neuropixels probes have 384 channels that are programmable across 960 sites. Their shanks are stable long-term in biological fluids or commonly used cell culture reagents, and their recording capabilities can last over a year, making them adequate for OI, from a materials standpoint.

At Johns Hopkins University, with funding from the NIH BRAIN Initiative, Harris' lab will finalize the family of recording probes with the Neuropixels 2.0 probes (Steinmetz et al., 2021), which are small enough to accommodate more than 10,000 channels in a freely moving mouse. They are smaller than the 1.0 version, and their geometry allows for the interface with organoids either by insertion into grown organoids or by using them as scaffolding, whereby the organoid would grow around the probe itself. While these Neuropixels probes are strictly for recording, the Neuropixels UHD would also be helpful for spike-sorting insights and can provide extra resolution from smaller sample sizes, potentially reading axons and/or dendrites.

Harris then provided alternative technologies to interface organoids such as silicon probes that are compatible with long-term imaging in the live brain and have a single unit resolution, although they are also rigid.

Tzahi Cohen-Karni, Associate Professor at the Department of Biomedical Engineering and Materials Science at Carnegie Mellon in Pittsburgh PA, presented a talk entitled “*Organ-on-electronic-chip forming input-output (I/O) with spheroids in 3D*.” Cohen-Karni's research bridges “soft and squishy” tissue that communicates *via* ions, with materials that are hard in nature, such as nanocarbons that communicate *via* electrons. He defined input as the ability to modulate cellular activity chemically or electrically and output as the ability to sense the electrical and chemical activity of electroactive cells.

His laboratory focuses on pushing the limits of the current platforms to enable long-term investigation and modulation of cellular electrical activity with high spatial and temporal resolutions and new types of hybrid nanomaterials for energy conversion, storage, and biosensing. In terms of spheroids, the driving force in developing three-dimensional entities is to record the activity of the spheroid, not just the bottom layer as would be the case with 2D systems.

Cohen-Karni introduced the Organ-on-e-Chip (Kalmykov et al., 2019), a robust microelectrodes array that is flexible and can be made porous for liquid and nutrient diffusion. Organ-on-e-Chip is capable of recording global and/or site-specific single channels, which makes it suitable for disease modeling, and while this model was originally used for cardiac spheroids, it has now also been used with cortical spheroids (Kalmykov et al., 2021). There is a need to develop multimodality platforms because electrical sensing is just on one end of the spectrum, highlighting the need for neurotransmitter detection with unique materials such as carbon fiber electrodes.

He stated that by using optical means to modulate electrical activity, we can send inputs to the tissue. Cohen-Karni's lab has created a broadband absorber to study the optical modulation of electrophysiology that generates heat when stimulated by light (Rastogi et al., 2020). This photothermal stimulation can be used to affect the organoid development as a cue, instead of genetic modifications. Cohen-Karni presented Mxene films, a new material that can be incorporated into cell cultures to form a hybrid of material and cell entity for I/O (Wang et al., 2021), and emphasized that “*Material science can help with I/O formation from network to single cell stimulation*.”

## Data analysis track introduction

Brian Caffo, a professor at the Department of Biostatistics, Johns Hopkins Bloomberg School of Public Health, gave a talk on “*Statistical methods for organoid intelligence*.” Caffo emphasized on the importance of repeatability and validity (statistical analysis of data repeatability measures), to ascertain that the methods are measuring what was intended.

He went on to say that we need efforts in source localization with sensors, e.g., electrodes, located at different distances, and variance estimates where uncertainties of localization are incorporated into the estimates. In describing signal summaries, Caffo went over the movement from simple task activation to more complex interactions, to brain mapping, then moving to clustering, connectivity, and unsupervised learning. From there, one moves to even higher complexity of epi-studies, dynamic connectivity, multimodal studies, and structure/function integration.

General patterns are observed in all computational biology, moving from lower to higher order interactions such as complex interactions, correlations and higher order moments, multiple modalities, time variation, larger scale studies, and robust application of machine learning. Finally, in terms of temporality and causality, Caffo believes the OI team will need to merge philosophical thinking on causality, temporal theories on causality, and modern graphical causal thinking.

Fang Han, an associate professor of Statistics and Economics at the University of Washington addressed AI analysis of OI. “*Our task at hand is to understand the relation between input and organoid changes (architecture/functionality), and to understand the relationship between organoid changes and output*.” Fang will develop a machine learning statistical model to separate meaningful outputs from the inputs. To do so, he explained, we will need “*machine learning, statistics, signal processing, information theory, and optimization. In the end, both deep learning and reinforcement learning will play key roles*.”

“*Where are we now?”* Han asked. “*What we have now are organoids caged in an integrated 2D-3D sensor, our input is the neurotransmitter glutamate, in terms of outputs, we have field potentials. What we wish to answer is what these changes mean in terms of functionality. How can we address this?”* Han explains that we can determine a simple threshold and monitor the heuristics spikes of firing strings of all the data before and after input. From this point, the structure, strength, and patterns of the data can be sorted, and from these data it is possible to analyze the location. To detect spikes and differentiate true signals from background noise, we can use machine learning/statistical tools such as intervention analysis, state-space models (decerns signal from noise), change-point detection, and combinatorial optimization. Then, pattern recognition can be implemented through intensity convolution models. Finally, we can investigate which parts of the organoid respond to the stimulus by using the sensor location paths and a mixture model. The organoid can be partitioned to a corresponding sensor, and the signal density received from the sensors can then be adjusted for distance. This way we can find the location in the organoid that is firing. “*We have a land of opportunity!.”*

Alex Szalay is the Bloomberg Distinguished Professor of Astronomy and Computer Science at the Johns Hopkins University, and architect for the Science Archive of the Sloan Digital Sky Survey. In his talk, “*Interactive Petabytes for Organoid Intelligence*,” he shared the lessons learned from 30 years of building large open public data sets and how to apply that knowledge to OI.

He illustrated a “big picture” of the emergence of big data by comparing past and present, highlighting that “*today's big science experiments such as the superconductor colliders, cost billions, take decades and may not be surpassed by another in our lifetime*.” In a new model, there is much more focus on generating and sharing novel data, and in terms of AI and machine learning, there is significant competition, hence the importance of agility vs. tenacity, where universities cannot compete with big industry (e.g., Google, Facebook), but can create unique, exciting data sets, and use AI for interpretation and discovery of novel patterns and phenomena. Szalay explained that this requires the data set to be AI-ready.

**Figure 2:**
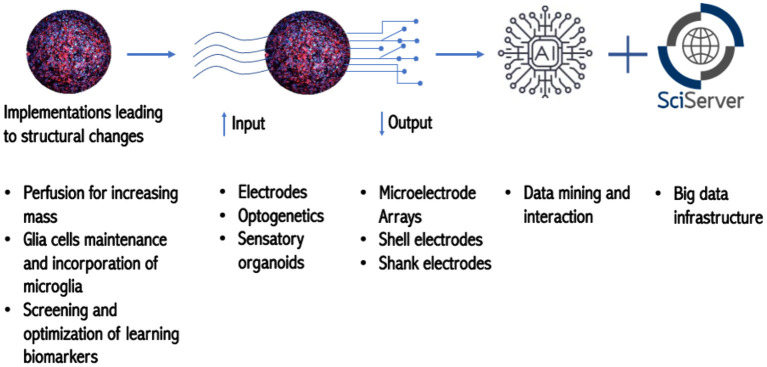
Roadmap toward OI.

In terms of data management and storage, Szalay introduced the Institute for Data Intensive Engineering and Science (IDIES), which promotes open science with interactive petabytes. They provide disruptive assistance from patterns to process and help projects at various levels of maturity. IDIES built the SciServer, which grew out of the platform they had constructed for astronomy. One of the main challenges of big data sets is data aggregation; and the utilization of interactive, collaborative use of petabyte-scale data potentially mitigates this problem. The SciServer is optimal for storing petabytes of data and has prompted collaborations with national labs and federal agencies such as National Aeronautics and Space Administration (NASA), National Institute of Standards and Technology (NIST), and of the Department of Energy (DOE) and other institutions such as the Max Planck Society. Furthermore, IDIES is equipped with 200 servers capable of storing more than 30 petabytes, which gives Johns Hopkins unique expertise to engage in the OI project (Figure 2).

### Challenges toward OI

The emergence of a new field naturally brings challenges. Many of these challenges, along with strategies to address them and move forward, were thematized in workgroup sessions during the second day, and summaries were reported on the third day of the workshop (Boxes 2–5).

Box 2 **Ethics track highlights.**

◦ Establishment of consistent terminology
◦ Public engagement and community-based approach
◦ Ethical, legal, and social implications (ELSI)
◦ International harmonization and governance
◦ Identification of overlapping frameworks for ethical and emerging technologies, what could be leveraged to help build OI framework, and what gaps need to be filled

Questions to be addressed:

1. Could brain organoids become conscious and/or sentient?
a. What is consciousness?
2. Could organoids experience pain and, if so, would they suffer – even in rudimentary ways? If so, at which stage of development?
3. Is there a moral obligation to inform the donor if, for example, something relevant to their health is identified during research?
4. Do donors have rights that extend beyond the donation e.g., novel uses/implications of their derived specimens beyond what was initially indicated in the consent form?
5. What are the technical, ethical, and legal boundaries for OI?

Box 3 **Organoids track highlights.**

◦ Cellular composition and reproducibility
◦ Microfluidics for perfusion, increasing mass and biocomplexity
◦ Specialized organoids network, e.g., connecting brain and retinal organoids
◦ Electrophysiology and imaging multiplexing
◦ 3D Shell MEA, MEA Mesh, and Neuropixels
◦ Optogenetics
◦ Organoid machine interface

Box 4 **E-physiology track highlights.**

◦ Improvement of biomaterials for long-term interface with organoids
◦ 3D Shell MEA, MEA Mesh, MEA sandwich, and Neuropixels
◦ Optogenetics
◦ Organoid-machine interface

Box 5 **Data analysis track highlights.**

◦ Machine learning
◦ Testable hypothesis
◦ Multimodal data analysis
◦ Type of data
◦ Standardized data structures and analysis
◦ Data storage
◦ Community based approach for moving forward

It is important to understand and reflect on the roots of why OI is being attempted. The first long-term goal is to improve societal outcomes by means of leveraging the advantages of biological computation, e.g., more energy efficiency, high-density storage, progressive learning, decisions based on incomplete datasets etc. Secondly, it is to understand how the human brain works by creating reductionist models of the human brain. Eventually, this could lead to understanding human disease and developing better treatments.

Across the four workgroups, the use of “consistent terminology” was discussed as being essential to enable clear communication across disciplines and public engagement.

## Ethics track

For OI a key question is whether capacities usually associated with human *beings*, such as intelligence and rationality, are, in fact, distinct from capacities typically associated with in silico systems, like computation? An entity meeting the aspirations of OI could risk breaching several foundational distinctions–organism and machine, living and non-living, human and non-human–that are widely used to make moral judgements. When one thinks about “intelligence,” it is likely that ideas of sentience and consciousness also arise. We need to establish proper terminology that gives serious consideration to how this terminology will impact listeners of all cultural and educational backgrounds (Paşca et al., 2022).

Recognizing the interplay between context, values, and beliefs will foster responsible dialogue with the public, and clear terminology and transparent communication regarding the nature and applicability of the research, i.e., medical, basic science, and/or commercial application, are essential to avert mis-interpretations of advances in OI that could inadvertently violate morally-relevant foundational distinctions. To complement these efforts, an international framework must be created that includes biosafety, neurodiversity, and international harmonization and governance. Finally, ethicists or scholars trained at the interface of neuroscience and society, need to be included in either all the grants or within each research team related to OI efforts.

We need to structure a principled baseline approach for OI with clear demarcations on what types of research and applications are allowed and what line of research, experiments, and applications should not be pursued. This effort can be supplemented by implementing a questionnaire asking about any potential negative consequences of OI-based research, to anticipate ethical, legal, and social implications. The integration of ethics at each step along the way, known as embedded ethics, is a pillar for OI and will be ongoing, and progress along with this emerging field. Embedded Ethics asks which of the technologies that we could create, *should* we create (Bonnemains et al., 2018; McLennan et al., 2022).

## Brain organoids track

How do 3D organoids differ from 2D cultures? From a computational and intelligence perspective, we need to decipher the connectivity systematically. The standards for organoids must be defined across fields, even when considering several lines of research. There is little to no requirements to document the basic characteristics of organoids when publishing; therefore, the establishment of standards directly affects benchmarking as we envision the reproducibility of this platform.

Defining “intelligence” in brain organoids depends on the endpoints being interrogated. These endpoints are constrained to what technology can measure and by the complexity of neuronal circuitry.

Electrophysiology, being a key readout of neural activity, can be used to screen whether the response (output) to a given stimulus (input) is altered in a reproducible way over time. But would predicting a response be considered intelligence or just adaptability? The expectation is that these stimulations and feedbacks lead to long-term potentiation (LTP), i.e., the process involving persistent strengthening of synapses that leads to a long-lasting increase in signal transmission between neurons. This LTP is a prerequisite of long-term memory.

The complexity of the central nervous system is established during embryogenesis, and several groups have shown that brain organoids recapitulate early stages of development (Lancaster and Knoblich, 2014; Trujillo et al., 2019). However, the complex topography of the human brain is formed in a protracted process of growth. Is this developmental maturity linked to intelligence? If so, how do we define a mature organoid? How do we improve their fitness and longevity?

Gyrification, or the folding process of the cerebral cortex, is not yet modeled in brain organoids. This critical process gives rise to the gyri and sulci, structures that separate brain regions and are linked to intelligence. These brain morphological and topographical features result from the expansion of the cortex (Menon, 2013; Herculano-Houzel et al., 2014), which is linked to increased size during evolution (Dunbar and Shultz, 2007; Hofman, 2014).

An ideal avenue to increase both mass and complexity is modeling vascularization in the organoids by perfusion. While these models exist, they are not advanced enough to provide perfusion to support a significant increase in organoid mass for increasing computational power. The implementation of a microfluidic system as a surrogate of vasculature would be able to support the metabolic needs of a growing organoid while patterning *via* microgradients. An added benefit is the homeostasis of culture conditions and the expected increased reproducibility of pattering. Initially, the costs of this platform will be high, but are expected to become more affordable as time passes, as is usually the case with any technology. It is, however, important to take full advantage of the intelligence/computing capabilities of current models. Simple 2D cultures have been trained to play pong (Kagan et al., 2022), and have controlled robotic navigation (Demarse et al., 2001).

### Technological roadmap

Measuring global electrical activity from the surface of the organoid is akin to human EEG systems and has been adapted to brain organoids in preliminary work (Huang et al., 2022). A fully embedded MEA mesh that integrates to the organoids during organogenesis, known as cyborg organoids (Li et al., 2019), is a possibility, as well as probing specific regions with high-resolution Neuropixels. These state-of-the-art technologies can provide valuable insights into trainability, computation, and learning capabilities.

Optogenetics, a technique that combines genetic engineering and optical technology to neuromodulate selected areas, can be applied to explore connectivity in organoids. This technique offers precise control of input on the cells of interest and is more specific than electrical stimulation. However, it also presents several challenges: the reporters can be toxic to the cells if overexpressed, the efficiency of transfection is highly variable, and cells may lose expression over time. Imaging remains an important challenge for multiplexing with electrophysiology to investigate region-specific electrical activity in response to stimuli. In addition to the difficulties of achieving high-resolution 3D reconstruction, long-term imaging can lead to phototoxicity. Functional magnetic resonance imaging (fMRI) could be an alternative to measure and map organoid activity.

## Electrophysiology track

One of the grand challenges discussed was how to best create well-defined problems to be solved by the brain organoid. Such a challenge can be based on the Modified National Institute of Standards and Technology (MNIST) database, which contains binary images of handwritten numbers. The MNIST database is commonly used in computer vision and machine learning. It is noteworthy that such problems have not yet been mapped onto and solved by 2D neuronal cultures.

We need to include other I/O modalities of biosensors to augment the capabilities of stimulating and recording. However, a potential roadblock for mapping functional connectivity is the speed at which optical and imaging platforms function. Once we understand the brain's structural and functional connections (the connectome), drug and toxicological tests on the organoid would provide valuable insight into understanding the impact of chemicals and drugs on functionality and how this may affect structure and circuitry.

While the idea of OI is futuristic and will face skepticism, we can make an analogy to the development of conventional computers, which circles back to standardization and benchmarking. Looking at the history of conventional computers, we see how originally, any makeshift part that worked was used, and the machines were very bulky. Soon, however, people made standards for the vacuum tubes and amplification ratio. Then, people made very specific modules like logic gates that were reproducible and could be used repeatedly in a predictable manner. It is not a stretch to think that as the field of OI comes into its own, organoids could be specialized for different areas of problem-solving. And eventually, more sophisticated systems that could work in parallel could be built.

More so, opportunities exist for realizing hybrid “bio-silicon” systems such as interfacing neural organoids with neuromorphic hardware, either for low-power biological computing or for neural rehabilitative purposes. For decades, researchers have been emulating neurophysiology in electronic integrated circuits from silicon neuron arrays, neuromorphic sensors such as event-based imagers, and silicon cochlea to actuators such as electronic central pattern generators among others. Interfacing such biologically plausible hardware with neural organoids could offer seamless bidirectional communication as both often speak the same language (spikes).

Such hardware may mediate information decoding and encoding between the organoid, its environment, and the existing digital computer, thereby facilitating practical deployment of organoids as useful computational engines. But even before that, consideration should be given to the neural interfacing setup to guarantee reliable I/O. This may be either invasive or non-invasive. Non-invasive options include optogenetic stimulation and recording such as calcium imaging, albeit at lower temporal resolution.

Another promising option for recording is Temporal Interference Stimulation and Impedance Tomography (TISIT) (Grossman et al., 2017). Temporal Interference Stimulation (TIS) involves the application of a pair of source-sink sinusoidal currents that differ in frequency by a relatively smaller margin to which the neurons respond. The underlying hypothesis is that the high frequency sinusoids are unlikely to independently activate neurons, but rather place them in refractory states. The low-frequency interfering electric field envelope, however, may suffice at eliciting neural activity. Regardless of the modality, a setup that preserves the spatial organization of the 3D organoid will be desirable. The organoid differentiation process involving constant gyratory shaking (Plummer et al., 2019) limits the feasibility of encapsulating organoids around electrodes during maturation. Thus, interfacing techniques post-differentiation are more suitable.

**Figure 3:**
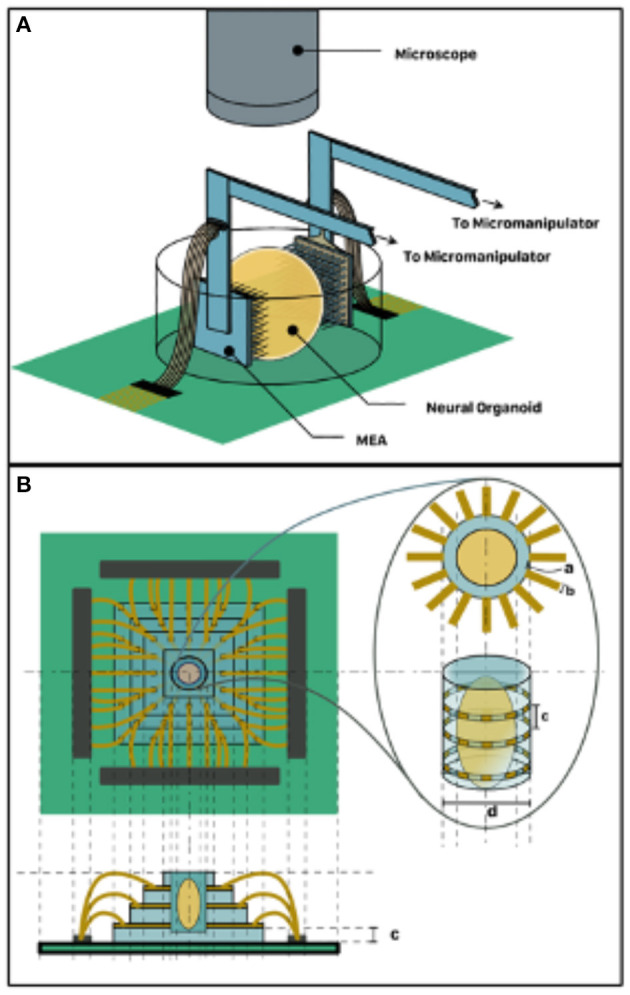
3D Organoid Interfacing. **(A)** MEA sandwich configuration–two MEAs assigned input and output respectively impinge the two organoid hemispheres. **(B)** Organoid well approach suitable for non-invasive interfacing such as TISIT.

An alternative to the self-folding electrode interfacing is the adoption of MEA sandwiching topology shown in Figure 3A. Here, a pair of medium- to high-density MEA such as the Utah Array can be micromanipulated to impinge the organoid *in vitro* on two sides, which could be designated as input and output. Another alternative is the organoid well approach, which involves the fabrication of a microfluidic glass-etched well with surrounding electrode sites shown in Figure 3B. This can be achieved by an initial step of bonding multiple glass layers bearing deposited electrode patterns. A well for holding the organoid can then be etched out of this stack. This will expose the cross-section of the electrode on the walls of the well and allow for non-invasive stimulation and recording. Similarly, fluid channels can also be etched to allow perfusion of the organoid with culture media. In both alternatives, the housing dish can be mounted on a custom printed circuit board (PCB) alongside neuromorphic and other electronics that mediate readout from the organoid to a digital computer. Neuromorphic hardware such as the Mihalas-Neibur Integrate-and-Fire Array Transceiver (MNIFAT) and other neuromorphic integrated circuits developed at the Etienne-Cummings lab can be adopted/ extend for such a mediation. Furthermore, memristive synaptic crossbar hardware can be mapped to the readout interface to facilitate training the organoid for use in a recognition task. An interesting direction to explore will be realization of generic Field Programmable Biological Assembloids (FPBAs) for purposes similar to Field Programmable Gate Arrays (FPGAs), where organoids can be repurposed for myriad computational functions not necessarily neurophysiological.

## Data analysis track

### Intelligence and self-organization

It will be necessary to explore the ways in which intelligence and self-organization arise at the cellular level and form networks and hierarchical organization. If we focus on what constitutes an intelligence/computation trait at the fundamental physical level, we should be able to study it structurally. But how can it be characterized? What kind of data is applicable to intelligence? How can we distinguish it from noise? Model making and predictions are two modes to characterize intelligence, and both can be viewed through a few existing theories such as active inference (Yufik et al., 2021), predictive processing (Ficco et al., 2021), free and energy minimization (Luhmann et al., 2019), or by the extension and derivation of other theories, e.g., embodied cognition (Mahon and Caramazza, 2008), self-organized critically (SOC) (Plenz et al., 1998), and dendric computing (Acharya et al., 2022). Thus, the architecture of brain organoids is of paramount consideration for achieving the organ(oid) function-level necessary to study intelligence and computation.

Nervous system research on intact and live zebrafish by non-invasive electrophysiology has shown spontaneous electrical activity of the brain and spinal cord (Tomasello and Sive, 2020); however, what this collection of spiking activity means remains unknown. We still do not know what this reflects or how they are processing inputs for a given output.

Understanding the connectome, the brain's structural and functional connections, is a promising area of research, especially as a function of time, and how it changes as a result of training. High-resolution imaging electron microscopy or 3D super-resolution live-cell imaging can help reveal connectivity structures related to functional outcomes. This information will also be highly relevant in addressing medical questions.

### Testable hypotheses

If we can find the “basis of intelligence” or identify a physiological threshold and establish a given value, it should be quantifiable and true across any mode of data input/output. Early understanding of the system as it grows and changes in complexity and establishing mechanisms of action are critical for exploring hypotheses to avoid the so-called “black box” pitfalls and spurious correlations. Coupling testable hypotheses with learning and neurocomputational theories, while creating benchmarks such as games and assays, can allow for the generation of key reproducible data.

OI has the potential to be generalized to human biology. Research from the Muotri group that used machine learning as an organoid age-predictor showed that cortical organoids are creating frequencies of electrical activity that mimic the prenatal up to postnatal human EEG signal (Trujillo et al., 2019).

### Multimodal data analysis

The aim is to make the OI findings replicable across a range of experimental environments (e.g., multimodal). From source localization, we can study hierarchical organization and the mapping of networks, including physical connections and synapse subtypes. In addition to electrophysiology, chemical, mechanical, optical and thermal I/O will also be analyzed and expanded to even noise and gravity in the future. The early consideration of compartmentalization of complex data systems into a simplified stream will facilitate data sharing with scientific communities across disciplines.

### Types of data

Units of data that can be done temporally, spatially, or both.

1. Single action potential
2. Spike trains
3. Synaptic activity/neurotransmitter recording
4. Population coding—Manifold spaces?
5. Connectivity between these data points
6. Metadata about the actual electrode maker, history of organoid including good cell culture practices, electrical properties of media, matrix properties, etc.
7. Input relative to all these

### Standardized data structures and analysis

When it comes to data analysis, the core question is how to glean biological meaning output from numbers and data input. It is critical to establish common and robust sets of data analysis approaches in order to satisfy the core question. Ideally, the analysis should be considered in conjunction with multiple sources of information and made available for people. A standardized open access data set can help catapult the involvement of the statistics community, such as those working with omics data sets. Furthermore, a standardized data curation can enable the development of shared tools and ensure reliability and confidence in OI data.

Finally, the establishment of a long-term, community-based approach to moving OI research forward is critical.

## Conclusions

The First Organoid Intelligence Workshop initiated the formation of an international and multidisciplinary community of scientists to establish OI as an ethical and scientific field of biological computing. The current technological state was discussed, as well as the challenges we need to overcome, and how to responsibly develop this burgeoning field in tandem with existing conventional and bio-inspired computing paradigms such as neuromorphic computing. The participants agreed on the Baltimore Declaration toward OI (Hartung et al., 2023), Box 1. Community formation is key for meeting the challenge of this emerging multidisciplinary field, and the participants suggested the regular repetition of this workshop to develop into a conference, and possibly into a scientific society. Furthermore, the creation of a dedicated journal was supported, though in the meantime, Frontiers in OI was established (#link). The capacity-building at Johns Hopkins University and beyond lends itself to the creation of a center and/or network to steer such developments. It will be fundamental to attract the resources for programs toward OI. Given that the technical elements seem to be within reach, this is an opportunity to be seized by the public and the private sector.

## Author contributions

IMP organized the workshop and drafted the paper. LS and TH developed the project. All authors contributed to the workshop discussions and the writing of the paper.

## Footnotes

^1^ https://mpsworldsummit.com/mps-world-summit-2022-3/

## Glossary

Big data–extensive datasets—primarily in the characteristics of volume, velocity, and/or variability—that require a scalable architecture for efficient storage, manipulation, and analysis (NIST, 2015).

**Biological computing–**tasks typically done by computers carried out by biological systems.

**Biosensors**–A biosensor is a device that measures biological or chemical reactions by generating signals proportional to the concentration of an analyte in the reaction (Bhalla et al., 2016).

**Cognition**–the human mental action or process of acquiring knowledge and understanding through thought, experience, and the senses (Dhakal and Bobrin, 2022).

**Connectome**–the brain's structural and functional connections in terms of networks (Leergaard et al., 2012; Contreras et al., 2015; Elam et al., 2021).

**Consciousness**–the human state of being aware of and responsive to one's surroundings (Oxford English Dictionary Oxford University Press, 2020); a hypothetical organoid's state of being responsive to and “aware of” the environment.

**Deep learning**–A type of machine learning, refers to artificial deep neural network, which is a specific configuration where neurons are organized in multiple successive layers, whereby increasing layers improves the power and performance of these methods (Chassagnon et al., 2020).

**Embedded Ethics**–the ongoing practice of integrating ethics into the entire development of a process as it progresses (Bonnemains et al., 2018; McLennan et al., 2022).

**Intelligence**–the human ability to acquire and apply knowledge and skills. Here, vision of OI-implementing cell models to perform computer functions (Merriam-Webster, 2022) allowing to test substances.

**I/O**–abbreviation for input/output.

**Long-term potentiation (LTP)**–a process involving persistent strengthening of synapses that leads to a long-lasting increase in signal transmission between neurons (Fu and Jhamandas, 2020).

**Memristive devices**–electrical resistance switches that can retain a state of internal resistance based on the history of applied voltage and current. These devices can store and process information, and offer several key performance characteristics that exceed conventional integrated circuit technology (Joshua Yang et al., 2013).

**Memory and Learning**– In the context of OI, learning is identified as an increased frequency to show and memorize a response pattern to a stimulatory pattern.

**Microelectrode Arrays (MEA)–**are cell culture dishes with embedded micro-electrodes that allow non-invasive measurement of neuronal network activity (Mossink et al., 2021).

Microphysiological systems (MPS)–also known as organoid or organ-on-a-chip technology, is an integrative microfabricated platform designed to recapitulate functional units of human organs (Bai and Wang, 2020).

M**ultimodal**–Incorporating or utilizing several different methods or systems (Oxford English Dictionary Oxford University Press, 2020). In the context of this manuscript is the integration and interpretation of data derived from a range of experimental environments and systems.

Neuropixels–Recording probes that can track the electrical activity of the same neurons over months (Kalmykov et al., 2019; Steinmetz et al., 2021).

**Optogenetics**–a technique that combines genetic engineering and optical technology to control and monitor biological functions (Joshi et al., 2020).

**Organoid intelligence (OI)**–describes an emerging field aiming to expand the definition of biocomputing toward brain-directed OI computing, i.e., to leverage the self-assembled machinery of three-dimensional (3D) human brain cell cultures (brain organoids) to memorize and compute inputs.

**Organ-on-chip**–are systems containing engineered or natural miniature tissues grown inside microfluidic chips (Leung et al., 2022)

**Reinforcement learning**–a computational approach to understanding and automating goal-directed learning and decision making. It is distinguished from other computational approaches by its emphasis on learning by an agent from direct interaction with its environment, without requiring exemplary supervision or complete models of the environment (Sutton and Barto, 2018).

**Self-organization**–The emergence of an overall order between parts of an initially disordered system that eventually give rise to higher ordered structures without the input of external forces (Lewis et al., 2010; Wedlich-Söldner and Betz, 2018).

**Sentience**–in humans, the simplest or most primitive form of cognition, consisting of a conscious awareness of stimuli without association or interpretation (Merriam-Webster, 2022); for OI, basic responsiveness to sensory input, e.g., light, heat etc.

**Synthetic intelligence**–Coined by John Haugeland, sometimes referred to as engineered intelligence, is a term derived from artificial intelligence. Refers to the synthesis of fundamental elements of intelligence as bona fide intelligence, and not just imitation (Law, 1994).
