# Supplementary material for: First Organoid Intelligence (OI) workshop to form an OI community
Source: Front Artif Intell. 2023 Feb 28;6:1116870. doi: 10.3389/frai.2023.1116870 (PMC10013972; doi:10.3389/frai.2023.1116870)
Supplement: Supplementary file 1 [file Data_Sheet_1.PDF]

First Organoid Intelligence Workshop

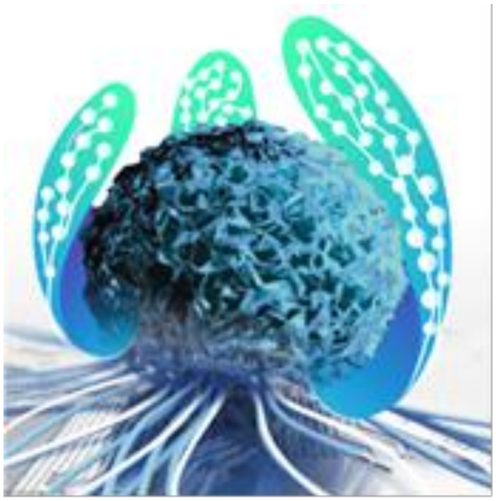

Forming an O.I. Community

February 22-24, 2022
Johns Hopkins University

Overview

The revolution in brain cell culture in 3D as organoids or organ-on-chip systems opens up for leveraging their brain functionality to study the physiology of learning and harness their capability of processing complex inputs to generate responses to control peripheral output devices. In short, we want to explore whether brain organoids can become biological computers. The expectation is that the combination with electrophysiology, high-content imaging, A.I. for pattern recognition as well as brain/sensory organoid and brain/machine interfaces will map the challenges and opportunities for biological computing.

Purpose

This three-day workshop will start with one-day of presentations from select invited multi-disciplinary experts from the ongoing feasibility study at Johns Hopkins, followed by a second day of discussion of four trajectories identified and next steps, and closing on the third day with an action plan. The feasibility study supported by WHO at Johns Hopkins will present its concept and preliminary results. This workshop is geared toward a consensus paper and a call on the scientific community to establish O.I. as a field. The workshop will bring together several experts to share their research experiences and discuss the opportunities and challenges of an O.I. approach.

CAAT Johns Hopkins University Center for Alternatives to Animal Testing
Johns Hopkins Whiting School of Engineering
i4 transatlantic think tank for toxicology
frontiers

Day 1 (Feb 22): Presentation Session

Time	Topics	Speakers
9:00 am – 9:05 am	**Welcome**	Dr. Frederick Fenter, Frontiers
9:05 am – 9:10 am	**Introduction & Vision**	Dr. Thomas Hartung, JHU
9:10 am – 10:00 am	**Session 1: The Ethics of O.I.**	
	Introduction to session	Dr. Jeffrey Kahn, JHU
	Talk 2: Stakeholder attitudes towards organoid research	Dr. Debra Mathews, JHU
	Talk 3: Towards responsible public engagement on neural organoids	Dr. Lomax Boyd, JHU
	Discussion	
10:00 am – 10:50 am	**Session 2: Organoids**	
	Talk 1: Development of human brain organoid technology	Dr. Alysson R. Muotri, UCSD
	Talk 2: Retinal organoids – human models of the sensory nervous system	Dr. Karl Wahlin, UCSD
	Talk 3: What do we need from biology to tackle O.I.?	Dr. Lena Smirnova, JHU
	Q & A	
Bio-Break		
11:00 am – 11:10 am	**Address by the Dean of the Whiting School of Engineering, JHU**	Dr. Ed Schlesinger, JHU
11:10 am – 12:00 pm	**Session 3: Electrophysiology**	
	Talk 1: Perspectives in integrated platforms for organoid-machine interfaces	Dr. David Gracias, JHU
	Talk 2: 3D Mesoscale Structures as Bioelectronic Interfaces to Cortical Spheroids	Dr. John Rogers, Northwestern University
	Talk 3: Considerations for high channel count Si probes as an electrophysiology probe for brain organoids	Dr. Tim Harris
	Talk 4: Organ-on-electronic-chip – forming I/O with spheroids in 3D	Dr. Tzahi Cohen Karn, Carnegie Mellon University
	Wrap-up	Dr. David Gracias, JHU
12:00 pm – 12:50 pm	**Session 4: Data Analysis**	
	Talk 1: Statistical methods for organoid intelligence	Dr. Brian Caffo, JHU
	Talk 2: Interactive Petabytes for O.I.	Dr. Alex Szalay, JHU
	Talk 3: A.I. analysis of organoid intelligence	Dr. Fang Han, University of Washington
	Q&A	
12:50 pm – 1:00 pm	**Discussion & Wrap-up**	Dr. Thomas Hartung, JHU

Day 2 (Feb 23): Discussion and workgroups		
**Time**	**Topics**	**Facilitators**
9:00 am – 9:30 am	**Introduction to Discussion Sessions, Tour de Table**	Dr. Thomas Hartung, JHU
9:30 am – 1:00 pm	**Session 1: The ethics of a "thinking human brain"**	Dr. Jeffrey Kahn, JHU
	**Session 2: Organoids**	Dr. Lena Smirnova, JHU
	**Session 3: Electrophysiology**	Dr. David Gracias, JHU
	**Session 4: Data Analysis**	Dr. Brian Caffo, JHU

Day 3 (Feb 24): Moving forward		
**Time**	**Topics**	**Facilitators**
**Call on the scientific community to create O.I. as a scientific discipline and action plans**		
9:00 am – 11:00 am	Reports from the four workgroups	Rapporteurs
11:00 am – 11:10 am	Bio Break	
11:10 am – 11:30 am	Frontiers journal and publication plans	Drs. Laure Sonnier and Lee Baker
11:30 pm – 12:55 pm	Toward O.I.: workshop paper, Declaration, community formation, initiating a project	Moderated by Dr. Thomas Hartung
12:55 pm – 1:00 pm	Wrap-up	

Meet the O.I. Speakers

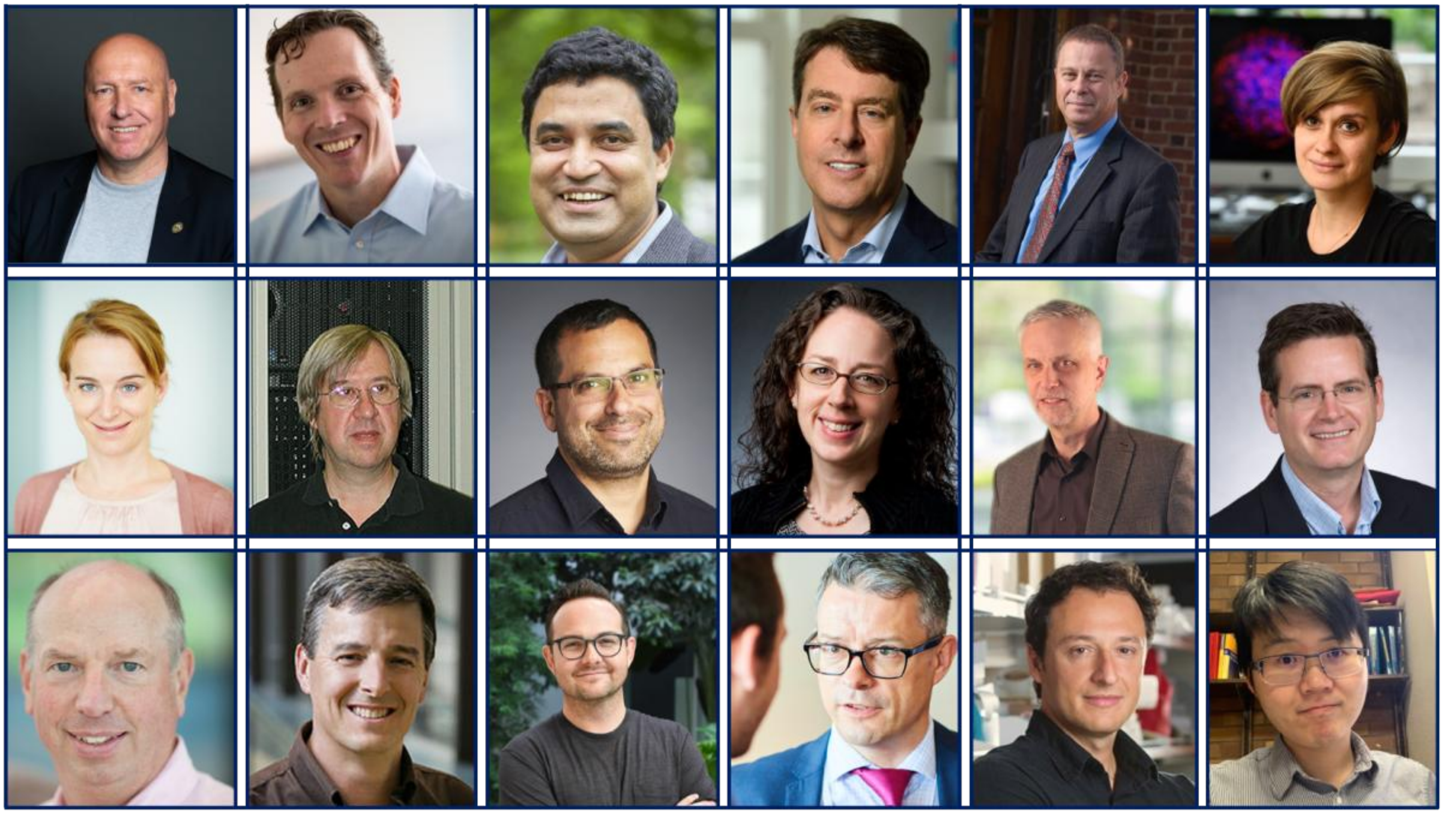

CAAT JOHNS HOPKINS UNIVERSITY Center for Alternatives to Animal Testing

JOHNS HOPKINS WHITING SCHOOL of ENGINEERING

t4 transatlantic think tank for toxicology

frontiers

Inaugural Speakers

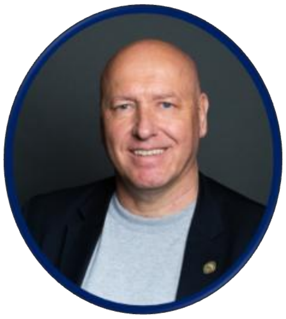

Thomas Hartung is the Doerenkamp-Zbinden-Chair for Evidence-based Toxicology in the Department of Environmental Health and Engineering at Johns Hopkins Bloomberg School of Public Health, Baltimore, with a joint appointment at the Whiting School of Engineering. He also holds a joint appointment for Molecular Microbiology and Immunology at the Bloomberg School. He is adjunct affiliate professor at Georgetown University, Washington D.C.. In addition, he holds a joint appointment as Professor for Pharmacology and Toxicology at University of Konstanz, Germany; he also is Director of Centers for Alternatives to Animal Testing (CAAT, http://caat.jhsph.edu) of both universities. CAAT hosts the secretariat of the Evidence-based Toxicology Collaboration (http://www.ebtox.org), the Good Read-Across Practice Collaboration, the Good Cell Culture Practice Collaboration, the Green Toxicology Collaboration and the Industry Refinement Working Group. As PI, he headed the Human Toxome project funded as an NIH Transformative Research Grant. He is Chief Editor of Frontiers in Artificial Intelligence. He is the former Head of the European Commission's Center for the Validation of Alternative Methods (ECVAM), Ispra, Italy, and has authored more than 600 scientific publications (h-index 100).

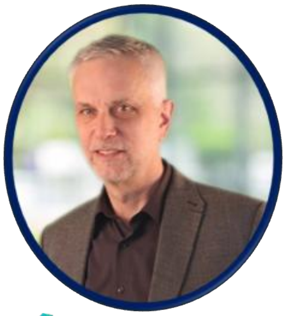

Frederick Fenter is the Chief Executive Editor of Frontiers, the open-access publisher based in Lausanne Switzerland. He studied chemistry (Ph.D. Harvard) and conducted research in atmospheric science for several years (h=16) before moving into scientific publishing with an appointment as manager of the Inorganic Chemistry Program at Elsevier Science, Lausanne. Since then, he has been founder of a start-up in publishing technology (FontisMedia SA, Lausanne); advisor to the launch of an institutional document repository (InfoScience); and publisher of an English-language University Press (EPFL Press, Lausanne). In 2013 he moved back to Frontiers, which he had previously advised as a consultant during its first 18 months of activity. As Chief Executive Editor at Frontiers, Fred oversees one of the largest editorial boards in scientific publishing, and leads third party negotiations, public affairs, and business development, among others. He also spearheads Frontiers' innovative editorial projects such as the Policy Labs, the Coronavirus Hub or the Frontiers Forum. An active advocate for Open Science, Fred frequently organizes and participates in a variety of advocacy events and roundtables.

Special Address

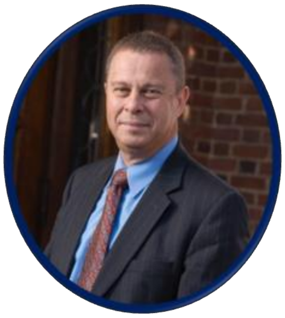

Ed Schlesinger is the Benjamin T. Rome Dean at Johns Hopkins University's Whiting School of Engineering where he also is a professor in the Department of Electrical and Computer Engineering. At Johns Hopkins, Schlesinger has launched numerous initiatives aimed at enhancing the student experience and the impact of the Whiting School of Engineering's educational and research efforts on society. He has built educational, research, and outreach partnerships and has enhanced translational opportunities for Whiting School of Engineering faculty, students, and staff. These efforts include the creation of the cross-divisional Department of Environmental Health and Engineering, a partnership between the Whiting School and JHU's Bloomberg School of Public Health. They also include the launch of the Malone Center for Engineering in Healthcare, a partnership with the School of Medicine, the Applied Physics Laboratory and other JHU divisions that is aimed at enhancing the efficiency, effectiveness, and consistency of health care, and helping lead a 10-year multimillion-dollar partnership between the Barclay Elementary/Middle School and JHU, community organizations, and the Baltimore City Schools to created pre-K through eighth grade school focused on engineering education and computer skills as a means to spark student achievement. Prior to joining Johns Hopkins in 2014, Schlesinger was the David Edward Schramm Memorial Professor and head of the Department of Electrical and Computer Engineering at Carnegie Mellon University, where he also served as the director of the Data Storage Systems Center, associate department head in ECE, founding co-director of the General Motors Collaborative Research Laboratory, and director the DARPA MISCIC Center. He has published over 250 articles and conference proceedings, and holds 13 patents. He is a Fellow of the IEEE and the SPIE, was President of the ECE Department Heads' Association and served on its board of directors, was a member of the International Advisory Panel for the A*STAR Graduate Academy in Singapore, was on the Advisory Board for the ECE Department at Georgia Tech and the Technology Commercialization Advisory Board for Innovation Works. He currently is a member of the Fellowship Evaluation Panel of the National Research Foundation of Singapore. Schlesinger earned a BSc in physics at the University of Toronto and earned his MS and PhD in applied physics at the California Institute of Technology.

Ethics Track

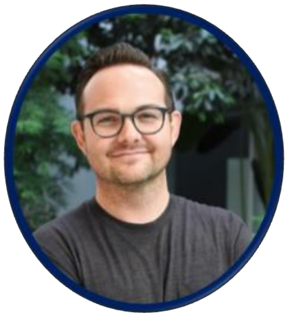

Lomax Boyd is a neurobiologist and creative producer serving as the Johns Hopkins Civic Science Fellow based at the Berman Institute of Bioethics and Kavli Neurodiscovery Institute. His research developing neuroscientific models to probe the evolutionary origins of the human brain has provoked curiosity and wonder about the brain, but also raised questions about how to seek, understand, and incorporate public input back into scientific research and technological advancement. Co-trained at the Center for Documentary Studies at Duke University, his creative practice seeks to explore how experimental, interactive, and traditional media can create new pathways for public engagement with science. He conducted his postdoctoral training in Erich Jarvis' Laboratory of Neurogenetics of Language at The Rockefeller University and received his PhD from Duke University investigating genetic basis of human brain size evolution. He has also held creative residencies as a Fulbright Scholar at the National Film Board of Canada and Science Education Fellow at the Howard Hughes Medical Institute.

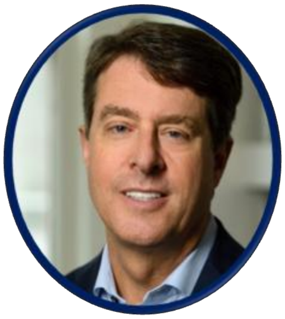

Jeffrey Kahn is the Andreas C. Dracopoulos Director of the Johns Hopkins Berman Institute of Bioethics, where he also holds the Robert Henry Levi and Ryda Hecht Levi Professorship in Bioethics and Public Policy. He is also Professor in the Dept. of Health Policy and Management of the Johns Hopkins Bloomberg School of Public Health. He works in a variety of areas of bioethics, exploring the intersection of ethics and health/science policy, including human and animal research ethics, public health, and ethical issues in emerging biomedical technologies. An elected member of the National Academy of Medicine, Prof. Kahn has served on numerous state and federal advisory panels; he is currently chair of the National Academies of Sciences, Engineering, and Medicine's Board on Health Sciences Policy, and has previously chaired its committee on the Use of Chimpanzees in Biomedical and Behavioral Research (2011) and the committee on Ethics Principles and Guidelines for Health Standards for Long Duration and Exploration Spaceflights (2014). In addition, Prof. Kahn was the founding president of the Association of Bioethics Program Directors, an office he held from 2006-2010. He is also an elected Fellow of The Hastings Center. Prof. Kahn's publications include Contemporary Issues in Bioethics; Beyond Consent: Seeking Justice in Research; and Ethics of Research With Human Subjects: Selected Policies and Resources, as well as over 125 articles. He speaks widely across the U.S. and around the world on a range of bioethics topics, in addition to frequent media outreach. From 1998-2002 he wrote the bi-weekly column Ethics Matters on CNN.com. Prior to joining the faculty at Johns Hopkins, Prof. Kahn was Director of the Center for Bioethics at the University of Minnesota. His education includes a BA in Microbiology (UCLA, 1983), MPH (Johns Hopkins, 1988), and PhD in Philosophy (Georgetown, 1989).

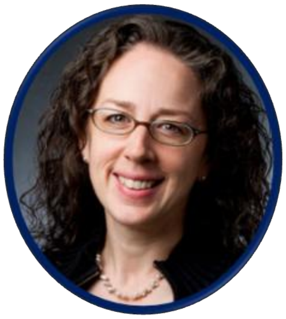

Debra Mathews is the Assistant Director for Science Programs for the Johns Hopkins Berman Institute of Bioethics, and an Associate Professor in the Department of Genetic Medicine, Johns Hopkins School of Medicine. Her academic work focuses on ethics and policy issues raised by emerging technologies, with particular focus on genetics, stem cell science, neuroscience, synthetic biology, and artificial intelligence. In addition to her academic work, Dr. Mathews has spent time at the Genetics and Public Policy Center, the US Department of Health and Human Services, the Presidential Commission for the Study of Bioethical Issues, and the National Academy of Medicine working in various capacities on science policy. Dr. Mathews earned her PhD in genetics from Case Western Reserve University, as well as a concurrent Master's in bioethics. She completed a Post-Doctoral Fellowship in genetics at Johns Hopkins, and the Greenwall Fellowship in Bioethics and Health Policy at Johns Hopkins and Georgetown Universities.

Organoids Track

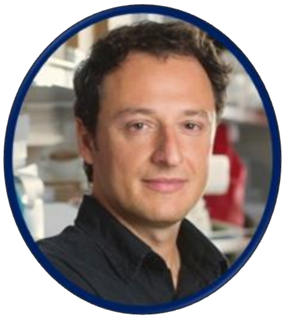

Alysson R. Muotri a professor in the Departments of Pediatrics and Cellular & Molecular Medicine at the University of California, San Diego, is focusing his research on solving one of life's greatest evolutionary and developmental perspective, differentiating stem cells to recreate "brain organoids" in the controlled setting of a lab. This work has implications for the generation of human disease models by determining the molecular and cellular mechanisms driving neurological complex disorders, such as autism. It is also creating opportunities for identifying and testing novel therapeutic approaches; the nature of this work reduces the amount of time required for moving new drugs to clinical trials. He obtained his B.S. in Biological Sciences and PhD in Genetics from the University of Campinas, Brazil and did his postdoctoral training on neuroscience and stem cells at the Salk Institute, La Jolla, CA.

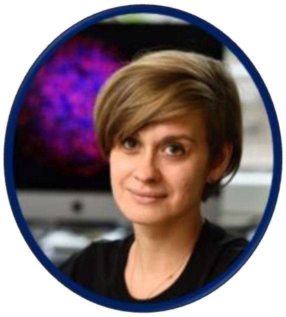

Lena Smirnova is a researcher at the Center for Alternatives to Animal Testing, at Johns Hopkins University, where she is leading the Education and Microphysiological systems programs, and Systems Toxicology. She has joint appointments at Johns Hopkins School of Engineering and George Town University. Her research focuses on the development of new approach methodologies for developmental neurotoxicity testing and understanding gene environmental interactions in autism. She received her PhD from Charite Free University, Berlin and her postdoctoral training from the Federal Institute for Risk Assessment, where she studied the role of microRNA in neural development, stem cell specification and developmental neurotoxicity. She is a Co-PI on a NCATS grant to organize a series of conferences on Microphysiological Systems (MPS) towards international MPS society.

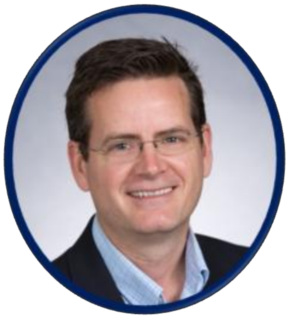

Karl Wahlin is an Assistant Professor in the Department of Ophthalmology at the University of California, San Diego. He received his doctorate from Johns Hopkins School of Medicine Department of Neuroscience in the laboratories of Drs. Ruben Adler and Donald Zack and completed his post-doctoral training at the Johns Hopkins in the Department of Ophthalmology where he specialized in differentiating human pluripotent stem cells (PSCs) into retinal neurons and RPE. His main research interests include retinal development, retinal degeneration and neuroprotection using pluripotent stem cell derived 'mini-retinas'. This work has broad implications in a wide range of retinal degenerative eye disorders including glaucoma, Leber's congenital amaurosis (LCA), Retinitis pigmentosa (RP), and age-related macular degeneration (AMD). His laboratory has also been developing gene-editing tools to engineer human PSC lines with fluorescent reporter activity to optimize in vitro models of human retinal development. Using patient derived hiPSCs and isogenic matched hiPSCs engineered with disease relevant mutations he is also developing human retinal disease models with which to devise strategies to block the disease process. A final area of interest is endogenous regeneration of photoreceptors through cellular reprogramming.

Electrophysiology Track

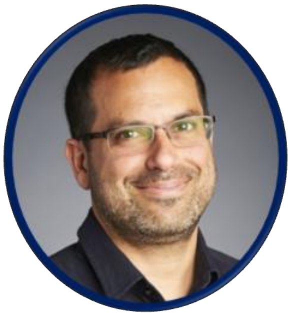

Tzahi Cohen-Karni is an Associate Professor at the Departments of Biomedical Engineering and Materials Science engineering in Carnegie Mellon University, Pittsburgh PA USA. He received both his B.Sc. degree in Materials Engineering and the B.A. degree in Chemistry from the Technion Israel Institute of Technology, Haifa, Israel, in 2004. His M.Sc. degree in Chemistry from Weizmann Institute of Science, Rehovot, Israel, in 2006 and his Ph.D. in Applied Physics from the School of Engineering and Applied Sciences, Harvard University, Cambridge MA, USA, in 2011. He was a Juvenile Diabetes Research Foundation (JDRF) Postdoctoral Fellow at the Massachusetts Institute of Technology and Boston Children's Hospital at the labs of Robert Langer and Daniel S. Kohane from 2011 to 2013. Dr. Cohen-Karni received the 2012 International Union of Pure and Applied Chemistry Young Chemist Award. In 2014, he was awarded the Charles E. Kaufman Foundation Young Investigator Research Award. In 2016, Dr. Cohen-Karni was awarded the NSF CAREER Award. In 2017, Dr. Cohen-Karni was awarded the Cellular and Molecular Bioengineering Rising Star Award, The Office of Naval Research Young Investigator Award and The George Tallman Ladd Research Award. In 2018, Dr. Cohen-Karni was awarded the Cellular and Molecular Bioengineering Young Innovator Award. In 2019, Dr. Cohen-Karni was awarded the Carnegie Institute of Technology (CIT) Dean's Early Career Fellowship.

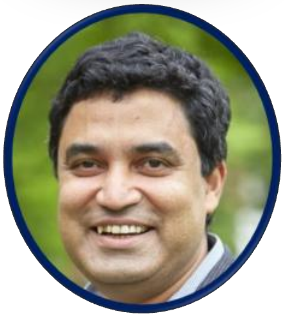

David H. Gracias is a professor at the Johns Hopkins University with a primary appointment in chemical and biomolecular engineering and secondary appointments in materials science and engineering, chemistry, and oncology (Johns Hopkins School of Medicine). He has published 195 technical articles and holds 33 issued patents in the areas of integrated circuit technology, self-assembly, self-folding and micro/nanotechnology. In 2018, building on over a decade of research on self-folding integrated platforms, Gracias and co-workers reported the development of shell microelectrode arrays (MEAs) and he is working with collaborators to develop integrated and self-folding microfluidic and MEA platforms for microphysiological and organoid systems. David is a Fellow of the IEEE, APS, AAAS, AIMBE, and RSC.

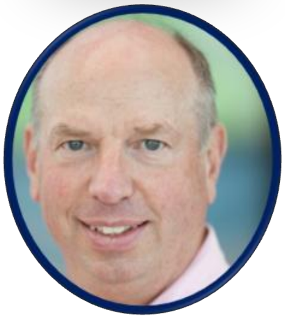

Tim Harris is a Group Leader and Senior Fellow at the HHMI Janelia Farm Research Campus in Ashburn, VA and a Research Professor at Johns Hopkins University Department of Biomedical Engineering. After his Ph.D. from Purdue University, Tim spent the following 18 very collaborative years at Bell Labs, Murray Hill, NJ. While at Bell, his work included high sensitivity fluorescence, the first use of CCD imagers for Raman scattering instrumentation, the characterization of quantum dots using a variety of spectroscopic tools, including fluorescence Near-Field Microscopy. In 1996 Harris moved to Seq Ltd. a small biotech startup in Princeton, NJ, where he led the development of high throughput multicolor confocal microscopy for cell-based drug screening, deployed to pharmaceutical labs around the world. Harris moved to Helicos Biosciences in January 2004 as the founding technical employee where his team reported the first single molecule DNA sequencing. In 2008 Tim moved to the HHMI Janelia Research Campus. Among others, he initiated a project, now called Neuropixels, jointly funded by HHMI, The Allen Institute for Brain Research, Gatsby Charitable Foundation, and the Wellcome Trust, partnered with Cambridge University and University College London. The resulting probes 384 channel fully integrated probes are now available. More than 6000 probes have been shipped to more than 600 labs since January, 2018. At Johns Hopkins, with funding from the NIH BRAIN Initiative, the Harris lab will finalize the family of recording probes with Neuropixels NXT, small enough for more than 10,000 channels in a freely moving mouse.

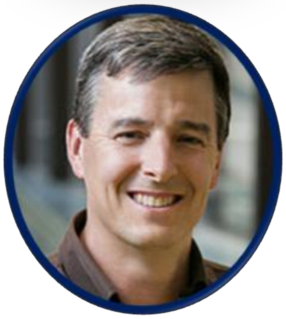

John Rogers began his career at Bell Laboratories as a Member of Technical Staff and then Director of the Condensed Matter Physics Research Department from 1997 to 2002. He then spent thirteen years on the faculty at University of Illinois, most recently as the Swanlund Chair Professor and Director of the Seitz Materials Research Laboratory. In the Fall of 2016, he joined Northwestern University where he is Director of the recently endowed Querrey-Simpson Institute for Bioelectronics. He is a member of the National Academy of Engineering, the National Academy of Sciences, the National Academy of Medicine and the American Academy of Arts and Sciences.

Data Analysis Track

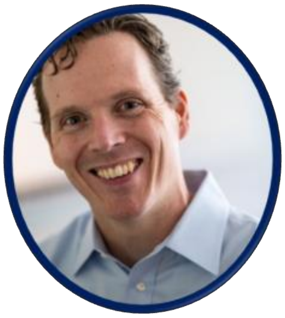

Brian Caffo received his doctorate in statistics from the University of Florida in 2001 before joining the faculty at the Johns Hopkins Department of Biostatistics, where he became a full professor in 2013. He has pursued research in statistical computing, generalized linear mixed models, neuroimaging, functional magnetic resonance imaging, image processing and the analysis of big data. He created and led a team that won the ADHD-200 prediction competition. He was the recipient of the Presidential Early Career Award for Scientist and Engineers, the highest award given by the US government for early career researchers in STEM fields. He co-created and co-directs the SMART (www.smart-stats.org) group focusing on statistical methodology for biological signals. He also co-created and co-directs the Data Science Specialization, a popular MOOC mini degree on data analysis and computing having over three million enrollments. Dr. Caffo is the former director of the graduate programs in Biostatistics, former faculty senate president and is the recipient of the Golden Apple teaching award and AMTRA mentoring awards.

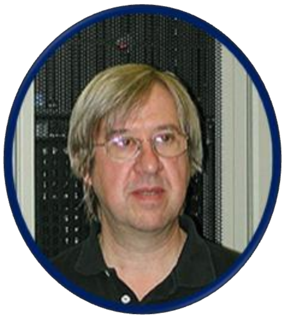

Alexander Szalay is the Bloomberg Distinguished Professor of Astronomy and Computer Science at the Johns Hopkins University. He is the architect for the Science Archive of the Sloan Digital Sky Survey. His papers span areas from astronomy, spatial statistics, computer science and more recently cancer research. He is a Corresponding Member of the Hungarian Academy of Sciences, and a Fellow of the American Academy of Arts and Sciences. In 2007 he received the Microsoft Jim Gray Award. In 2015 he received the IEEE Sidney Fernbach Award for his contributions to Data Intensive Computing. In 2020 he was awarded the Victor Ambartsumian International Science Prize for his work in physical cosmology. In 2021 he and his team were recognized with the ACM SIGMOD Systems award.

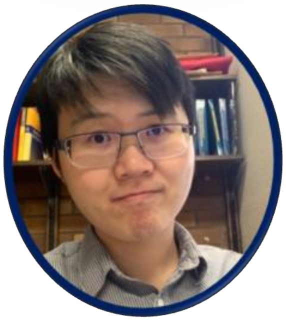

Fang Han received his Ph.D. from the Department of Biostatistics, Johns Hopkins University in 2015. He is currently an Associate Professor of Statistics and Economics at the University of Washington. His main research interests are in rank- and graph-based statistics, statistical optimal transport, mixture models, nonparametric and semi-parametric regressions, time series analysis, as well as their applications to complex biological, social, and neuroscience data. His research has been featured in a number of top journals and conferences spanning a variety of fields including statistics (the Annals of Statistics, JRSSB, JASA, Biometrika), economics (Journal of Econometrics), probability (Electronic Journal of Probability), information theory (IEEE Transactions on Information Theory), biology (Nature Molecular Psychiatry), and machine learning (NeurIPS, ICML). He is the recipient of the 2021 Bernoulli Society New Research Award that recognizes the work of outstanding young researchers in mathematical statistics biannually.

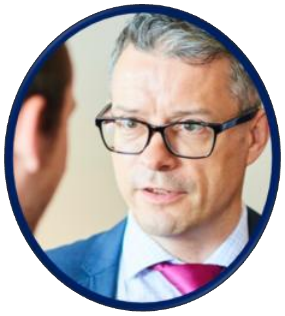

Lee Baker is the Editorial Adviser to Frontiers in Science. Lee has worked for over 25 years in scientific and science-policy communications in health sciences. After working as a Clinical Pharmacist in the UK health service he moved into medical communications industry in 1995 as a writer and editor supporting the pharmaceutical industry and academia in all forms of bespoke peer-reviewed and standalone publications relating to preclinical and clinical drug research and development. Since 2000 he has worked in parallel as a consultant helping leading researchers, patient organizations, scientific societies, and industry innovators to inform health and research/innovation policy at the European level, including through multidisciplinary and multistakeholder initiatives and strategic publications translating science into policy recommendations in numerous fields.

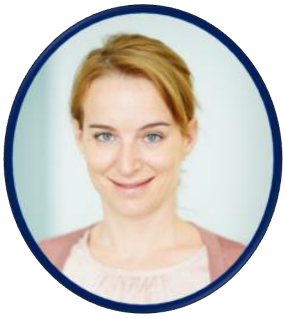

Laure Sonnier is the Executive Director of Frontiers in Science. She is responsible for journal strategy and execution oversight. Laure holds a PhD in Neuroscience from Ecole Normale Supérieure (Paris, France) and a Master of Public Health from Harvard University. Laure spent 8 years condu cting research on the development of the vertebrate nervous system in the laboratory of Dr. Alain Prochiantz (Ecole Normale Supérieure, Collège de France) fostering further collaborations with the laboratories of Professor Wolfgang Wurst and of Professor Magdalena Götz (Max Planck Institute, Münich) as well as Professor Martin Hirsch (La pitié Salpétrière, Paris, France). After post-graduate studies at Harvard, Laure transitioned her professional career to public affairs and public policy working at the interface of science, health and policymaking. Laure has spent the past 12 years in Brussels working within the European Policy ecosystem coordinating public affairs and strategic engagement programs for various stakeholders and constituencies within the health and research fields.
